# Supplementary material for: Synthesis of Densely Immobilized Gold-Assembled Silica Nanostructures
Source: Int J Mol Sci. 2021 Mar 3;22(5):2543. doi: 10.3390/ijms22052543 (PMC7961356; doi:10.3390/ijms22052543)
Supplement: Supplementary file 1 [file ijms-22-02543-s001.pdf]

Synthesis of Densely Immobilized Gold-assembled Silica Nanostructures

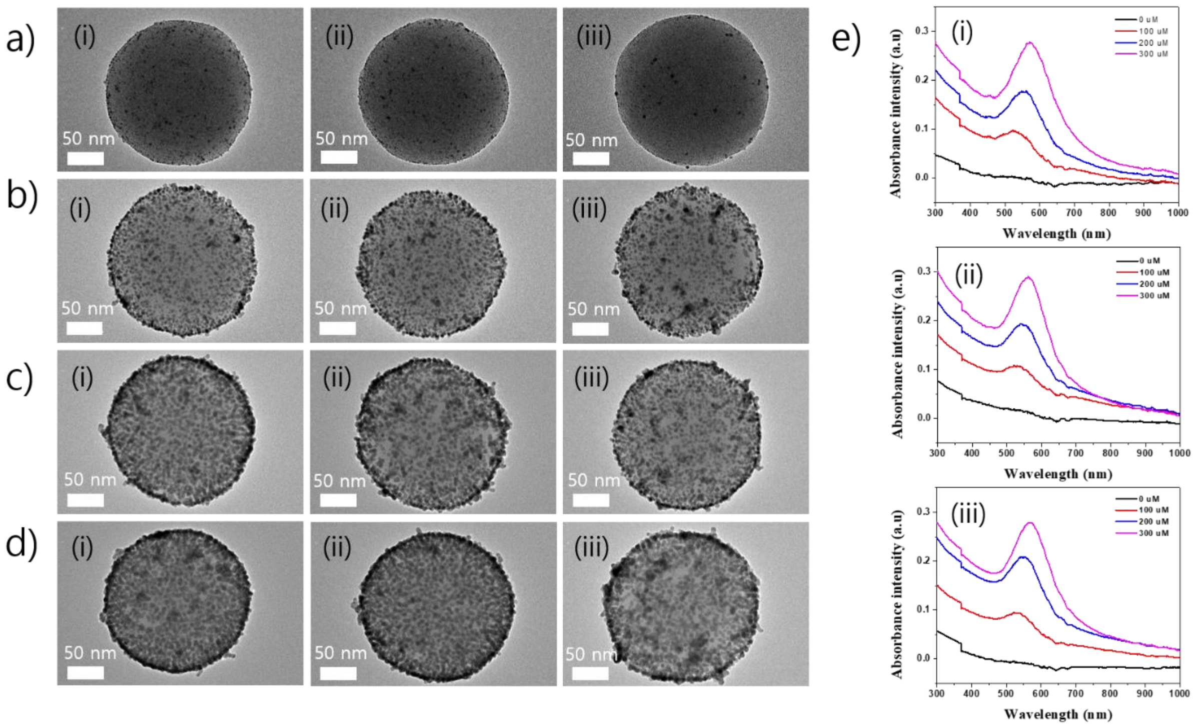

Figure S1. TEM images and UV-Vis spectra of SiO_2@Au nanostructures synthesized at (i) 2 mg (ii) 5 mg and (iii) 10 mg SiO_2@NH_2 in (a) 0, (b) 100, (c) 200, and (d) 300 μM Au^{3+} concentration.

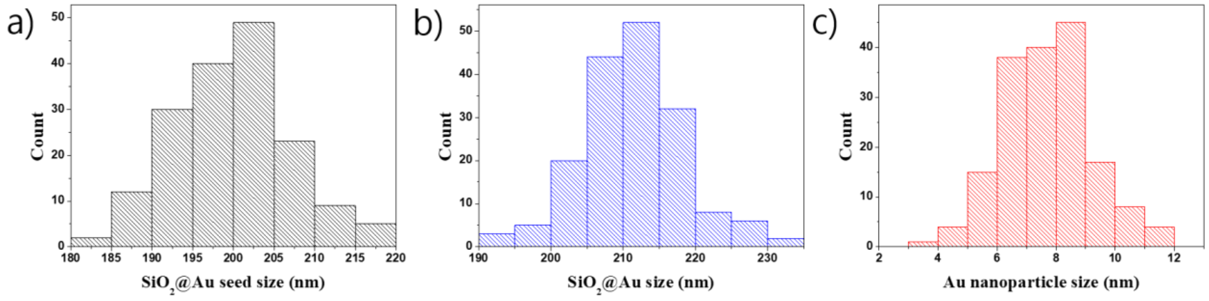

Figure S2. Histogram of nanoparticle size: (a) SiO_2@Au seed (n = 170), (b) SiO_2@Au synthesized at 150 μM Au^{3+} (n =172) and (c) HF treated-SiO_2@Au (n=172).

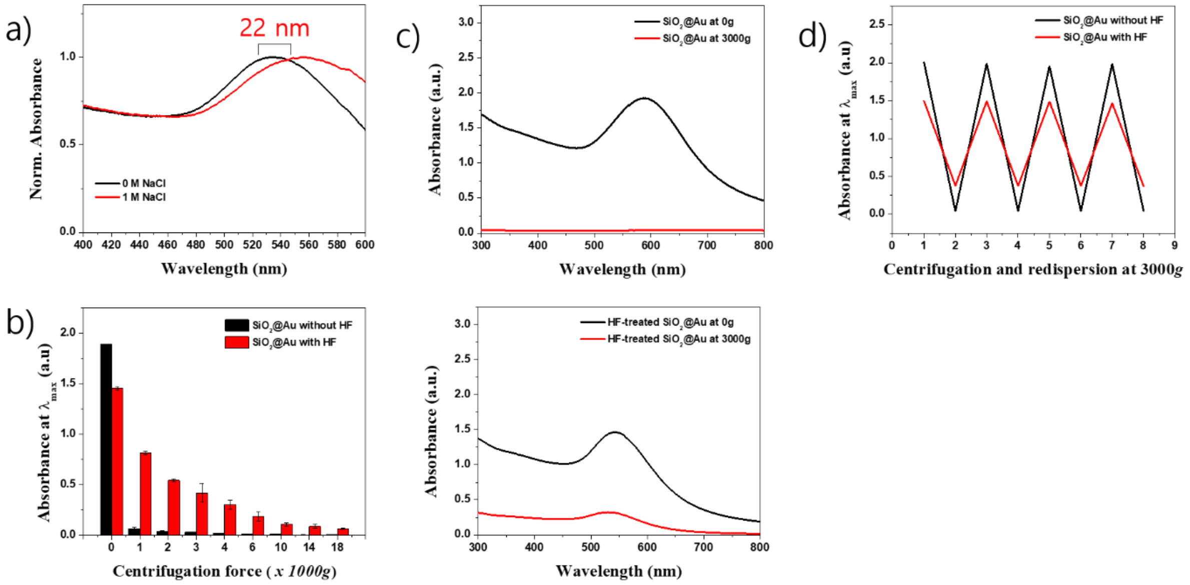

Figure S3. a) Red-shifting of UV-Vis spectra of SiO_2@Au treated HF, (b) effect of centrifugation speed, (c) UV-Vis spectra of SiO_2@Au and HF-treated SiO_2@Au, (d) optical absorbance plot of SiO_2@Au nanostructures synthesized at 150 mM Au^{3+} with and without HF treatment after centrifugation (2, 4, 6, 8 in x-axis) and redispersion (3, 5, 7 in x-axis).

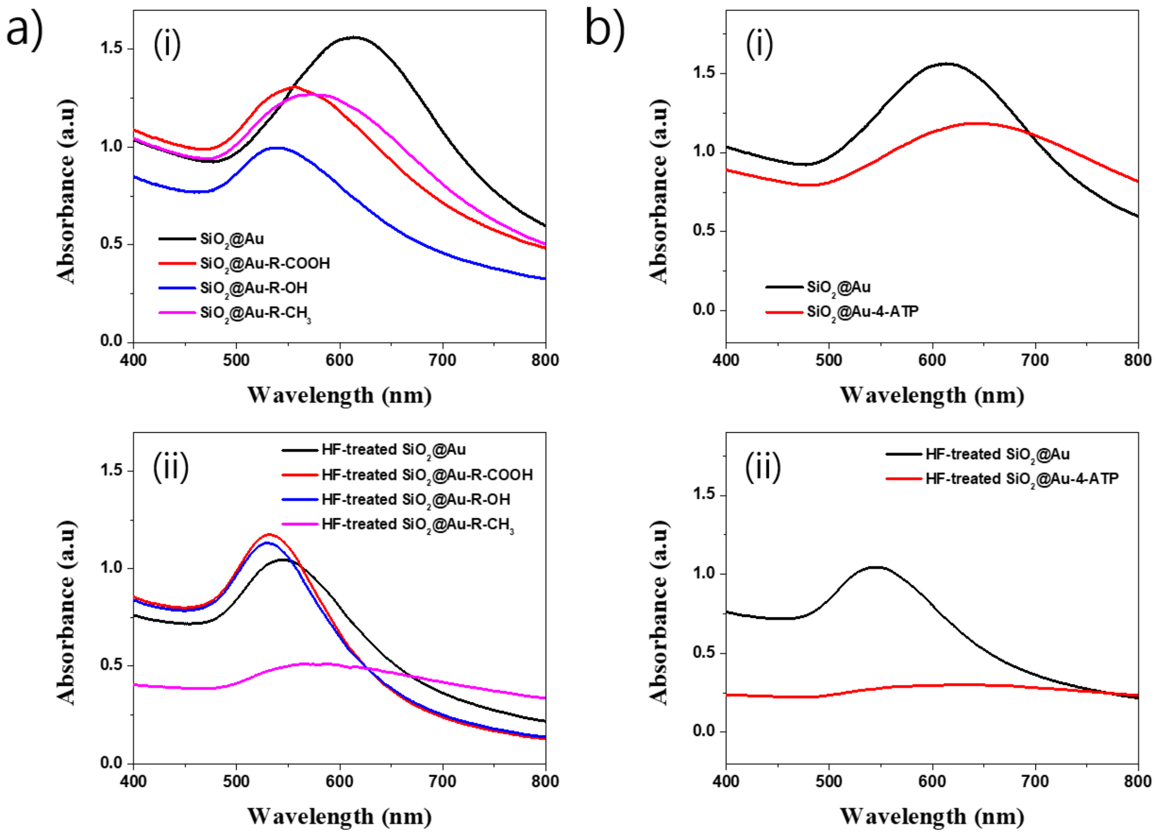

Figure S4. Effect of surface modification of (a) 1-undecanethiol (R-CH_3), 11-mercaptopundecanoic acid (R-COOH) and 11-mercapto-1-undecanol (R-OH) ligands and (b) 4-ATP on the UV-Vis spectra of (i) SiO_2@Au nanostructures and HF-treated SiO_2@Au synthesized at 150 mM Au^{3+}.

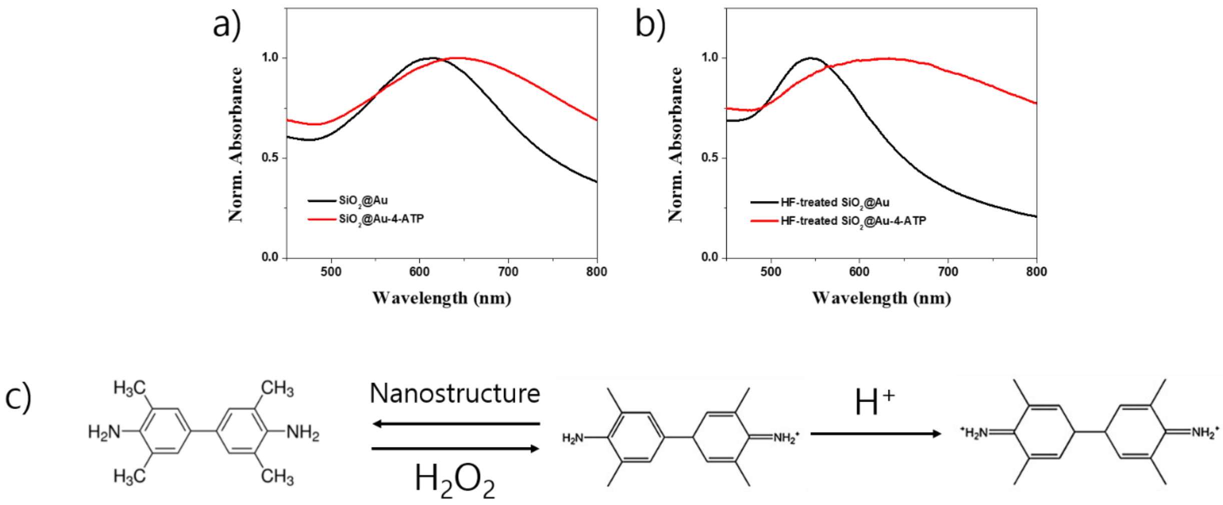

Figure S5. Normalized absorbance spectra of (a) SiO_2@Au nanostructures and (b) HF-treated SiO_2@Au synthesized at 150 mM Au^{3+}. (c) Mechanism of peroxidase-like catalytic activity of SiO_2@Au nanostructures.
